# Effects of Carbonaceous Materials with Different Structures on Cadmium Fractions and Microecology in Cadmium-Contaminated Soils

**DOI:** 10.3390/ijerph191912381

**Published:** 2022-09-28

**Authors:** Zihan Long, Chunya Ma, Jian Zhu, Ping Wang, Yelin Zhu, Zhiming Liu

**Affiliations:** 1 College of Environmental Science and Engineering, Central South University of Forestry and Technology, Changsha 410004, China; 2 Longyou Ecological Environmental Protection Agency, Quzhou 324400, China; 3 Longyou Ecological Environment Monitoring Station, Quzhou 324400, China; 4 Department of Biology, Eastern New Mexico University, Portales, NM 88130, USA

**Keywords:** biochar, graphene, carbon nanotubes, cadmium, microecology

## Abstract

Carbonaceous materials have proved to be effective in cadmium remediation, but their influences on soil microecology have not been studied well. Taking the structural differences and the maintenance of soil health as the entry point, we chose graphene (G), multi-walled carbon nanotubes (MWCNTs), and wetland plant-based biochar (ZBC) as natural and engineered carbonaceous materials to explore their effects on Cd fractions, nutrients, enzyme activities, and microbial communities in soils. The results showed that ZBC had stronger electronegativity and more oxygen-containing functional groups, which were related to its better performance in reducing soil acid-extractable cadmium (EX-Cd) among the three materials, with a reduction rate of 2.83–9.44%. Additionally, ZBC had greater positive effects in terms of improving soil properties, nutrients, and enzyme activities. Redundancy analysis and correlation analysis showed that ZBC could increase the content of organic matter and available potassium, enhance the activity of urease and sucrase, and regulate individual bacterial abundance, thereby reducing soil EX-Cd. Three carbonaceous materials could maintain the diversity of soil microorganisms and the stability of the microbial community structures to a certain extent, except for the high-dose application of ZBC. In conclusion, ZBC could better immobilize Cd and maintain soil health in a short period of time.

## 1. Introduction

Soil is an important part of the ecosystem and provides necessary conditions for human survival and development. However, soil cadmium (Cd) pollution has become a global problem which threatens the sustainable use of soil resources and the safe cultivation of food. Gorospe et al. [1] analyzed 16 different heavy metals in 91 vegetable-garden soil samples in San Francisco, USA. The results exhibited that the number of gardens with heavy metal concentrations was higher than the local human health screening level, exceeding 75%. Kumar et al. [2] collected and sorted data for heavy-metal soils in India from 1991 to 2018, and the results showed that the average Cd value of all soil types exceeded the limit [2]. In China, Cd accumulation in soil has been found in more than 11 provinces and 25 districts [3]. According to the statistics, in China, there was nearly 2.6 × 10^7^ hm^2^ of cultivated area contaminated by heavy metals, with approximately 40% of the soil polluted by Cd, leading to almost 1.46 × 10^8^ kg of cereal crops being contaminated by Cd from 2006–2014 [4,5]. Consequently, remediation strategies are urgently required to control Cd in soils for crop security.

Among various conventional remediation methods, an in situ immobilization technique with exterior amendments has been proposed as an effective approach to reduce the accumulation of Cd in crops and the bioavailability of Cd in soil [6,7,8] which could meet the technical needs of the safe utilization of moderately and mildly polluted farmland soil in China. Generally, the common soil amendments include inorganic passivators, represented by calcareous minerals; clay minerals and metal oxides; and organic passivators, represented by biochar and organic fertilizers [9]. Among these, carbonaceous materials are the subject of wide attention because of their abundant sources and low costs. Moreover, with stable carbon atoms as the basic framework, carbon materials have high chemical stability, high temperature resistance, and excellent electrical and thermal conductivity. Carbon fiber, mesoporous carbon, fullerenes, graphene, and carbon nanotubes are commonly used carbonaceous materials in the environment.

Biochar (BC), as a kind of carbonaceous material with a fine-grained porous graphite microcrystalline structure, has been widely studied as an amendment for Cd remediation [10,11,12]. Abbas et al. [13] pointed out that rice-straw biochar could decrease bioavailable Cd in soil and alleviate Cd-induced toxicity in wheat, and this effect was more significant under a higher dose of rice-straw biochar [13]. Some studies have also affirmed that BC has a positive effect on the enhancement of the nutrition and water-holding capacity of soils [14,15,16]. Furthermore, previous studies have reported that BC could increase numbers of Gram-positive and Gram-negative bacteria, supplement unstable carbon substrates, and dissolve fatty carbon, thus improving the activities of soil microorganisms [17,18,19].

Graphene (G) and carbon nanotubes (CNTs) are two common engineered carbonaceous nanomaterials. G is a two-dimensional carbon-based nanomaterial composed of sp^2^ hybrid carbon atoms, and CNTs are one-dimensional carbon-based nanomaterials that consist of single- and multi-layer cylindrical hexagonal graphene curls. Due to their versatile and specific properties, more attention has been paid to their application in controlling heavy-metal pollution, especially in the removal of metals from aqueous solutions [20,21,22]. Although some researchers have found that carbonaceous nanomaterials could also be feasible and innovative correction tools for immobilizing heavy metals [23,24], their effects on soil microecology are poorly understood. A study found that microbial biomass was scarcely affected by the application of GO [25]. When the concentration of MWCNTs exceeded 5 mg∙g^−1^, soil microbial biomass was significantly reduced. When the concentration was lower than 1 mg∙g^−1^, the soil microbial community composition and enzyme activity could be stable for a short period of time.

Both natural carbonaceous materials (biochar) and engineered carbonaceous materials (MWCNTs and graphene) have been reported to be effective in reducing the bioavailability of Cd, thus making them promising amendments for Cd remediation. However, the application of such carbonaceous amendments may have some unexpected results, such as changes to soil microbiology. To our knowledge, most researchers have adopted pure cultures to explore the toxicological effects of nanocarbon materials but have paid little attention to the microbial communities in soils. Although the effect of biochar on soil microorganisms has been widely studied, the possible relationships between soil microorganisms and biochar properties have not been explained. Moreover, few studies have systematically explored the short-term effects of carbonaceous amendment by comparing natural carbonaceous materials with engineered carbonaceous materials. Thus, the purpose of this study was: (1) to examine the effect of carbonaceous amendments (natural and engineered) with different structures on the fractionation of Cd in soils and (2) to investigate the impacts on soil enzyme activity, microbial community, and soil nutrients of applications of carbonaceous amendments under different doses. This work is expected to enhance our understanding of the use of carbonaceous amendments with various structures and at different doses for Cd remediation from the perspective of soil health.

## 2. Materials and Methods

### 2.1. Carbonaceous Materials and Soil

The natural carbonaceous material (biochar) used in this study was derived from Thalia dealbata Fraser., a wetland plant which was collected by the Yanghu Reclaimed Water Co., Ltd., Changsha, China. The plant samples were washed, placed in the air to dry at room temperature, then ground through a 10-mesh sieve. A quantity of 30 g of plant powder was added to a crucible with a lid. The crucible was placed in a muffle furnace (SX-4-10, Tester, Tianjin, China) and heated at a rate of 15 °C·min^−1^ under a limited supply of oxygen. Samples were preheated at 100 °C for an hour and then pyrolyzed at a final temperature of 500 °C, with a retention time of 2 h. The prepared biochar was named ZBC.

Graphene (G; diameter: 0.5–5 μm, thickness: 0.8–1.2 nm, purity: >99% wt.%) and multi-walled carbon nanotubes (long) (MWCNTs; long: 10–30 μm, diameter: 10–20 nm, purity: >95% wt.%) were purchased from Nanjing XFNANO Materials Tech Co., Ltd. (Nanjing, China).

Cd-contaminated soil samples were collected from the surface layer of a pilot rice field (Liling, China) using a five-point sampling method. In order to remove plant material and stones from the soil, the samples were air-dried, ground, and passed through a 2.0 mm sieve before the incubation experiment. Table S1 shows the main physical and chemical properties of the experimental soil samples.

### 2.2. Experimental Design and Sample Collection

The air-dried, sieved soil samples (200 g) were placed in 500 mL plastic beakers. Before adding amendments to the soils, the samples were preincubated at 25 °C in the dark for one week using an illuminating incubator (LRH-400-G, Shaoguan Taihong, Shaoguan, China) to stabilize the microbial activity. Then, the contaminated soils were well-mixed with ZBC at different concentrations (0.5%, 1%, and 5%) and with G powder and MWCNT powder at different concentrations (0.01%, 0.05%, and 0.1%) on a dry-soil basis, while soil without amendments was used as the control (CK). All the plastic beakers were laid in an illuminating incubator at 25 °C for 30 days in the dark. Three replicates for each treatment were run. During the incubation period, samples were weighed every 5 days, and deionized water was added to 60% of the field-water-holding capacity of the soils to maintain constant moisture contents. The treated soils were air-dried when the incubation was over and then ground into powder and passed through specific meshes (2.0 mm or 0.15 mm) for further analysis.

### 2.3. Characterization

The morphologies of the G, MWCNTs, and ZBC were characterized by scanning electron microscopy (SEM; JSM-6380LV, Shimadzu, Japan). Specific surface areas were tested through Brunauer–Emmett–Teller analysis (BET; JSM-6380LV, Quadrasorb SI, Conta, Boynton Beach, FL, USA), and N2 was used as the adsorption gas. The infrared spectrum was determined using a Fourier transform infrared spectrometer (FTIR-650, Shanghai Precision Instruments, Shanghai, China) to obtain the relevant information on the active groups of the samples.

### 2.4. Soil Sample Analysis

The pH levels, moisture contents, organic matter (OM) contents, cation-exchange capacities (CECs), and soil nutrients, such as alkali-hydrolyzed nitrogen (AN), available potassium (AK), and available phosphorus (AP), of the soil samples which had been passed through a 2.0 mm sieve were analyzed. Each sample was dispersed with deionized water (1:2.5) for 1 h, then pH was measured with a high-precision pH meter (PHS-3C, Leici, Shanghai, China). The moisture content was determined by comparing the quality of the soil before and after drying (105 °C, 24 h). The OM contents and CECs of the soil samples were measured by oxidation according to the potassium dichromate colorimetric and ammonium acetate methods, respectively [26,27]. AN, AK, and AP were evaluated by the alkali-diffusion method, ammonium acetate extraction-flame photometry, and molybdenum blue colorimetry, respectively [28].

The air-dried subsamples which had been passed through a 0.15 mm sieve were digested with HNO_3_-HClO_4_ (*v*/*v*: 4:1) to analyze the total Cd contents [29]. The fractions of various defined Cd species were determined through the BCR extraction method [30] and could be divided into four forms: acid-extractable (EX-Cd), oxidizable, (OX-Cd), reducible (RD-Cd), and residual fractions (RS-Cd).

Acid phosphatase (ACP), sucrase (SUC), catalase (CAT), and urease (Urea) are common indicators used to evaluate soil enzyme activity. According to the previous detection methods described by Dick et al. [31], phosphophenyl disodium colorimetry, 3,5-nitrosalicylic-acid colorimetry, potassium permanganate titration, and phenol–sodium hypochlorite colorimetry were used to analyze the above enzyme activities.

### 2.5. DNA Extraction and PCR Reaction

DNA extraction and quantification was an important part of the project. The equipment used in this study included a FastDNA TM SPIN Kit for Soil (Waltham, MA, USA) and a NanoDrop 2000 (Waltham, MA, USA) [32]. The amplification by polymerase chain reaction (PCR) and sequencing of the gene fragments of the bacterial 16s RNA were completed by Biomarker Technologies (Beijing, China), using the Illumina MiSeq 2500 sequencing platform (Illumina, San Diego, CA, USA).

### 2.6. Sequencing Analysis and Statistical Analysis

The QIIME 1.8.0 toolkit was used to analyze the taxonomic and diversity indices [32]. Operational taxonomic units (OTUs) were defined at the 97% similarity level by UCLUST [33]. Four indices, including ACE, Chao1, Simpson, and Shannon, were generally adopted to estimate α-diversity. Based on Bray–Curtis distances, taxonomic- and phylogenetic-based β-diversity indices were evaluated. Using the “PCoA” function in R (version 3.3.1, Robert G. and Ross I., UoA, NT), the changes in bacterial community structure were explored [34]. Bray–Curtis difference analyses between samples yielded cluster analysis results. Redundancy analysis (RDA) was conducted using CANOCO 4.0 to explore the relationship between soil quality and bacterial community. After testing the normality and variance homogeneity, one-way analysis of variance (ANOVA) was performed to analyze the relationships between the various indices. Where the treatment effect was significant (*p* < 0.05), a Duncan’s test was performed to test the significance (α = 0.05) for pairwise comparisons.

## 3. Results

### 3.1. Characterization of Carbonaceous Amendments

**Figure 1:**
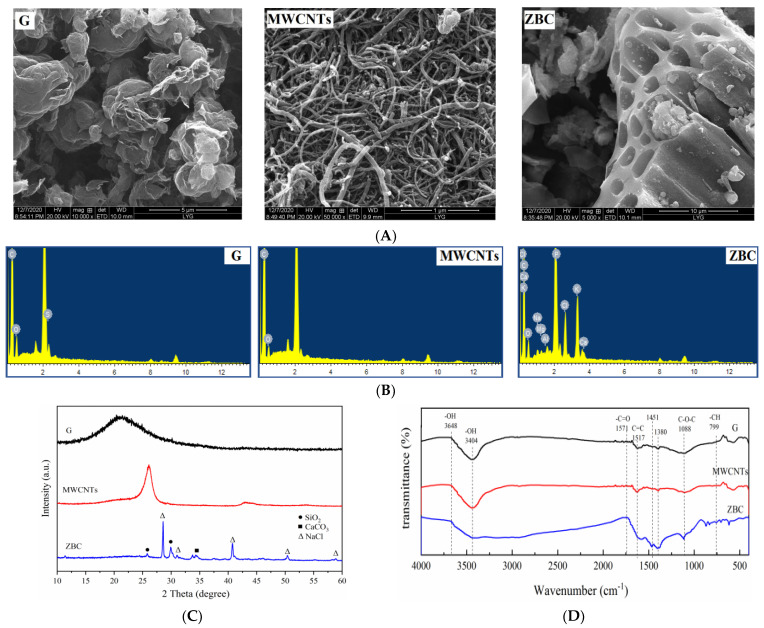
(**A**) SEM, (**B**) EDS (Full scale 1282 cts cursor: 0.000), (**C**) XRD, and (**D**) FTIR results for G, MWCNTs, and ZBC. G: graphene; MWCNTs: multi-walled carbon nanotubes; ZBC: biochar derived from a wetland plant (Thalia dealbata Fraser).

SEM images revealed that G had a lamellar structure, whose white part was the curl of the edge of graphene. MWCNTs had long, curved, thin nanostructures. ZBC had a better pore structure, and the pore holes appeared on the surface (Figure 1A). The element composition on the surface of ZBC was more abundant than that of G and MWCNTs (Figure 1B). In addition to the shared elements C and O between the three carbonaceous amendments, ZBC also had unique elements, such as P, K, Ca, Na, Mg, and so on. The XRD results showed that G displayed a strong, sharp peak at around 2θ = 22.48°, which corresponded to a d-spacing of 0.395 nm. MWCNTs possessed a prominent peak at around 2θ = 26.01°, which corresponded to a d-spacing of 0.176 nm. ZBC exhibited more diffraction peaks, suggesting that the crystal structure of ZBC was more complex. Obvious peaks for SiO_2_, CaCO_3_, and NaCl could be observed. Additionally [35], the characteristic peak of carbon disappeared near 2θ = 23.0°, indicating that pyrolysis at high temperatures could destroy the carbon-fiber crystal structure of the biochar (Figure 1C). The infrared-spectrogram-curve trends of the three carbonaceous amendments were generally similar, indicating that each material contained abundant surface functional groups, such as the stretching vibration of -OH (3000–3700 cm^−1^), the stretching vibration of the double bonds of C=C and -C=O (1500–1800 cm^−1^), and the stretching vibration of -COOH and -CHO (1300–1500 cm^−1^). The contents of functional groups were different. For example, compared with ZBC, G and MWCNTs had stronger -OH-stretching-vibration peaks at 3648 cm^−1^ and 3404 cm^−1^. In addition, the stretching vibration of the -C=O double bond at 1751 cm^−1^, the stretching vibration of the C=C double bond at 1517 cm^−1^, and the stretching vibration of -COOH, -CHO at 1300–1500 cm^−1^ in ZBC were stronger than those of G and MWCNTs. Moreover, the BET surface area and the hole volume of G were the largest, followed by those of MWCNTs and ZBC (Table S2). The BET surface areas of the G and MWCNTs were 127.3 times and 4.8 times that of ZBC, respectively. ZBC had the largest hole diameter and strongest electronegativity.

### 3.2. Effects of Carbonaceous Amendments on Basic Soil Properties and Cd Fractionation

**Figure 2:**
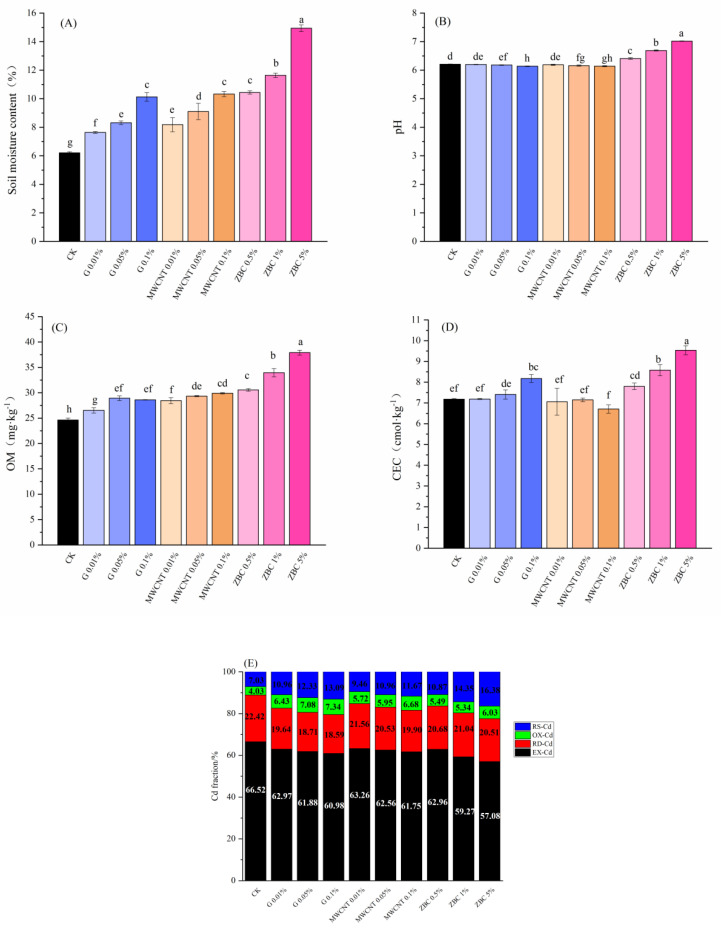
Effects of carbonaceous amendments on (**A**) moisture content, (**B**) pH, (**C**) OM (organic matter), (**D**) CEC (cation exchange capacity), and (**E**) Cd fractionation in the soils (n = 3). Different lowercase letters in the same column indicate significant differences between the treatments (*p* < 0.05), and the same applies hereafter.

The application of G, MWCNTs, and ZBC could increase soil moisture content and OM, and ZBC was more efficient in improving them (Figure 2A,C). G and MWCNT treatments slightly reduced the soil pH, while ZBC significantly increased the soil pH by 0.2–0.79 units (*p* < 0.05, Figure 2B). Different from G and the ZBC group, soil CEC was reduced by 0.42–6.65% under MWCNT treatment (Figure 2D). In our tested soil, Cd mainly existed in the form of EX-Cd, followed by RD-Cd, OX-Cd, and RS-Cd. With increases in G, MWCNTs, and ZBC, the EX-Cd contents decreased by 3.55–5.54% (G), 3.26–4.77% (MWCNTs), and 3.56–9.44% (ZBC), and increases in RS-Cd could also be observed (Figure 2E).

### 3.3. Effect of Carbonaceous Amendments on Soil Microecology

#### 3.3.1. Soil Nutrients

The three carbonaceous amendments could increase AN contents, except for the 0.1% MWCNT group. The highest AN contents were observed for the 0.01% MWCNT, 0.01% ZBC, and G 0.05% groups (Figure S1A). Unlike G and MWCNTs, ZBC could increase AP content (Figure S1B). Additionally, with increases in G, MWCNTs, and ZBC, AK contents increased (Figure S1C).

#### 3.3.2. Soil Enzyme Activity

After incubation, CAT activity was reduced by G and MWCNTs, while it increased after ZBC application (Figure S2A). The ZBC 5% treatment resulted in the highest Urea activity, but for G and MWCNTs, an exceeding dosage (>0.05%) resulted in reductions in Urea (Figure S2B, *p* < 0.05). G and MWCNTs had little effect on ACP (*p* > 0.05), while ZBC significantly decreased ACP, with a reduction of 18.10% under the 5% treatment (Figure S2C, *p* < 0.05). With increases in G, MWCNTs, and ZBC, SUC activities remarkably increased by 12.27–24.07% (G), 4.46–16.86% (MWCNTs), and 116.24–188.34% (ZBC), respectively (Figure S2D).

#### 3.3.3. Effect of Carbonaceous Amendments on Soil Microbial Communities

##### Bacterial Community Diversity

**Figure 3:**
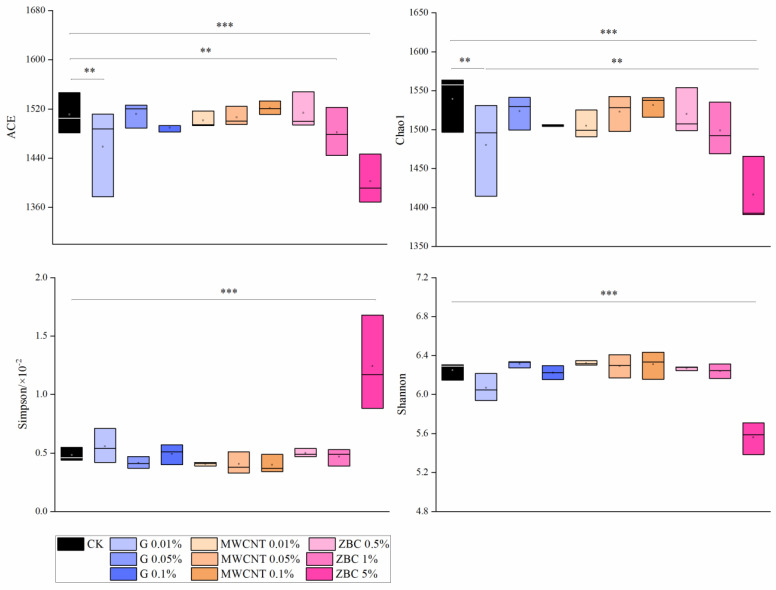
Effects of carbonaceous amendments on α-diversity index. (** *p* < 0.01, *** *p* < 0.001, and the same applies hereafter).

The rarefaction curve showed that the OTU number was in the range of 1230–1513 and gradually reached a plateau, indicating that the microbial community in the soil sample had been completely covered (Figure S3). After applying different doses of G, MWCNTs, and ZBC to the soil, the overall diversities did not undergo any significant changes (Figure 3; *p* > 0.05). Under the ZBC 5% treatment, the ACE and Chao1 indices were significantly reduced, while the Simpson index increased significantly, indicating that applying too much biochar would reduce the abundance and uniformity of soil microbial diversity.

##### Bacterial Community Composition and Structure

Proteobacteria, Acidobacteria, Chloroflexi, Bacteroidetes, Gemmatimonadetes, and Actinobacteria were the dominant species under all treatments, accounting for more than 80% of all sequences (Figure S4). Different microbes responded differently to the various carbonaceous amendments. For example, the RAs of phyla (Proteobacteria and Chloroflexi) decreased in comparison with CK after the introduction of G, MWCNTs, and ZBC, but the differences were not significant (*p* > 0.05). Slight increases in the RAs of the phylum Acidobacteria were found in the nanocarbon-material-treated samples and the low-concentration ZBC-treated samples.

There were various processed samples distributed in each area of the data space (Figure S5), including 29.40% of the total variance belonging to PC1 and 21.08% to PC2. The distance between the CK and ZBC 5% treatments was great, indicating that biochar at a higher dosage could change the composition of bacterial communities. With the increase in ZBC, the bacterial community structure moved to the right along the PC1 axis and moved upward along the PC2 axis, suggesting that ZBC had a significant dose effect on microbial community structure.

##### Correlation between Bacterial, Soil Nutrient, and Enzymatic Activities

Shannon and Chao1 were used to characterize the α-diversity of the soil bacteria, and NMDS1 and NMDS2 represented the β-diversity. The results showed that bacterial diversity had stronger relationships with soil enzyme activity and nutrients in the ZBC group. For example, β-diversity was significantly positively correlated with soil Urea, SUC, AP, and AK and significantly negatively correlated with ACP and AN (Table S3; *p* < 0.01). Additionally, under the ZBC treatment, more bacterial phyla (such as Proteobacteria, Acidobacteria, Bacteroidetes, Patescibacteria, and Rokubacteria) had significant positive or negative correlations with soil enzyme activity and soil nutrients (Figure S6).

##### Correlations between Environmental Parameters and Microbial Communities

**Figure 4:**
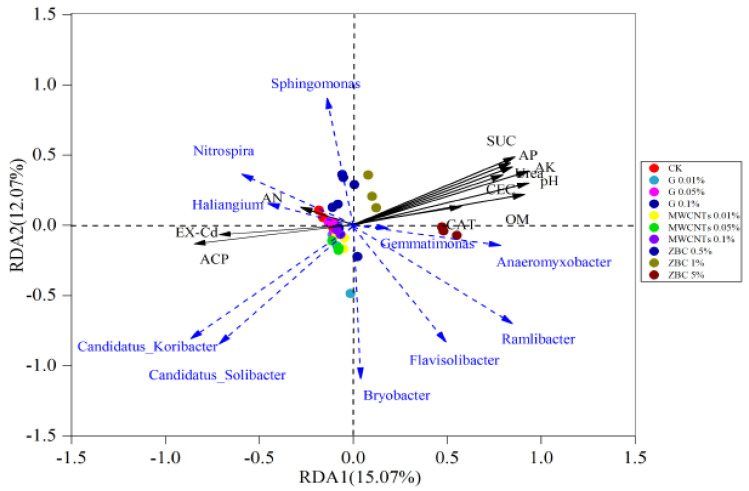
RDA analysis of soil biochemical properties and soil microbial structure at the genus level.

RDA analysis suggested that pH, AK, AP, SUC, and ACP were the main factors involved in the microbial community changes (Figure 4). The content of EX-Cd had a negative relationship with pH, OM, CEC, AP, AK, SUC, Urea, and CAT and a positive correlation with AN and ACP. Moreover, EX-Cd content had a positive correlation with the RAs of Nitrospira, Sphingomonas, Haliangium, Candidatus_Koribacter, and Candidatus_Solibacter and a negative relationship with Gemmatimonas, Anaeromyxobacter, Ramlibacter, Flavisolibacter, and Bryobacter. The results indicated that the G, MWCNT, and ZBC treatments could reduce the proportion of EX-Cd by increasing the contents of OM and AK and the activities of SUC and Urea (Figure 1A and Figure 2, Tables S2 and S5). On the other hand, the introduction of G, MWCNTs, and ZBC increased the abundances of Flavisolibacter, Bryobacter, and Anaeromyxobacter and reduced Nitrospira and Haliangium, thus reducing the content of EX-Cd.

## 4. Discussion

### 4.1. Effect of Carbonaceous Amendments on Cd Fractions

Assessing the risk of heavy metals from total contents was not quite comprehensive because not all forms have the same impact on the environment [29]. In our study, the addition of G, MWCNTs, and ZBC could reduce EX-Cd and increase RS-Cd in the soil (Figure 2E), suggesting that these carbonaceous amendments could effectively immobilize Cd; ZBC was the most efficient.

ZBC had more abundant functional groups, better pore structure, and higher electronegativity [36] than G and MWCNTs (Figure 1A and Table S2). A significant positive correlation between Zeta potential and EX-Cd was observed (Table S4, *p* < 0.01, R^2^ = 0.755), indicating that the stronger electronegativity of ZBC played an important role in reducing EX-Cd. Previous studies have pointed out that the strong electronegativity of biochar renders it a high cation-exchange capacity and thus the capability to fix heavy metals [36]. Figure 1D also showed that ZBC possessed richer oxygen-containing functional groups, which were beneficial for the immobilization of Cd by providing heavy-metal binding sites to form complexes [37].

ZBC could better reduce Cd by regulating the basic physical and chemical properties of soils (such as increasing soil pH, CEC, and OM). In the present study, G, MWCNTs, and ZBC increased soil moisture content and OM (Figure 2A,C). The improvement in soil moisture content was mainly due to the nano-level particle sizes of G and MWCNTs and the highly porous structure of ZBC. OM was an important factor in immobilizing heavy metals by complexation, ion exchange, and adsorption. In our study, the content of OM increased by 24.16–54.10% in the ZBC group, approximately four times as much as in the G and MWCNT groups. We presumed that the reason was the differences in the availability of carbon. Compared with biochar, the carbon structures in G and MWCNTs were more stable and harder to utilize. The main factors that controlled Cd adsorption were CEC and pH [38]. ZBC input could increase soil pH (Figure 2B) because the negatively charged functional groups on its surface could combine with H+ ions in the soil. However, pH decreased mildly under the G and MWCNT treatments. This was likely due to the negative charge of the outer membrane and the emergence of many active centers created by its own cage aromatic structure, large surface steps and the roughness of nanocarbons, and the serious mismatch of bond states [7]. Additionally, soil CEC increased under the G and ZBC treatments, while it decreased under MWCNT treatment (Figure 2D, *p* < 0.05). The increase in CEC under ZBC treatment was probably due to the abundance of the elements K, Ca, and Mg, which could increase the numbers of exchangeable ions. Furthermore, in the process of the oxidation of aromatic compounds on the surface of biochar to form carboxyl functional groups, the adsorption value of cations increased. Moreover, correlation analysis results also showed that there was a clear negative correlation between pH, CEC, OM, and EX-Cd under ZBC treatment (Table S5, *p* < 0.01), the response of basic soil physical and chemical indices to EX-Cd being stronger than under G and MWCNT treatments.

It Is generally known that biochar can enhance capacities for heavy-metal immobilization by changing physicochemical characters, whose mechanisms are mainly chemical interactions [39,40]. However, knowledge of the resistance and control mechanisms of carbon nanomaterials in relation to soil Cd is still lacking. Some researchers have reported that decline in Cd bioavailability may be related to surface cation-exchange mechanisms and adsorption mechanisms [41,42]. Combined with the larger BET surface areas, we considered that G and MWCNTs immobilized Cd mainly via physical adsorption mechanisms, which is coherent with the results obtained by Yang et al. [43].

### 4.2. Effect of Carbonaceous Amendments on Soil Microecology

#### 4.2.1. Soil Nutrients

Essential elements for plant growth and development usually include nitrogen, potassium, and phosphorus. In this study, the application of low doses of nanocarbon materials and ZBC increased contents of AN (Figure S1A) because the nitrogen carried by them entered into the soil. The reduction in AN under ZBC 5% was probably related to the volatile loss of ammonia with the increase in pH. An increase in AP under ZBC treatment was observed (Figure S1B), which was consistent with previous studies [44,45]. However, the negatively charged ions carried by G and MWCNTs would compete with the PO43 ions in the soil for the specific adsorption sites on the surfaces of the soil particles, thus reducing the ability of soil to adsorb phosphorus. The three carbonaceous amendments could all increase soil AK contents, with the ZBC group having the highest AK content (Figure S1C). This was mainly related to ZBC’s own potassium content.

#### 4.2.2. Soil Enzyme Activities

Soil enzyme activities are important indicators of soil health and can be used to evaluate the effects of heavy metals on soil microbial activities [46,47]. In this study, the influences of carbonaceous amendments on enzyme activities in the contaminated soil were dependent on the different types of amendments and enzymes. The three carbonaceous amendments could significantly increase Urea and SUC activities (Figure S2). However, 1% MWCNTs slightly decreased Urea activity. The CAT activity increased slightly under ZBC treatment, whereas it decreased with the application of G and MWCNTs. The employed G and MWCNTs did not apparently affect ACP activity, except for the remarkable decrease in ACP observed in the ZBC group. Previous studies have reported that the enhanced activity of soil enzymes due to biochar is probably due to the reduction in the bioavailability of heavy metals and their toxicity to microorganisms [43,48], which could help to explain the promotional effects of CAT, Urea, and SUC activities in this study. In addition, the decreased ACP activity after the ZBC application was related to the increase in pH. As reported, the optimum pH was 4.0–5.0 for ACP [46]; thus, the improvement of pH may result in a shift in phosphatase activity to neutral phosphatase activities. Moreover, the stimulation of enzyme activities by G and MWCNTs was likely due to the immobilization of the enzymes [49]. However, G and MWCNTs reduced CAT activity, indicating that such carbonaceous nanomaterials tend to reduce soil resistance to the environment to some extent. Wei and Ge [50] reported that the protein skeleton of CAT would be unfolded and lost in the presence of GO, thus decreasing CAT activity after G and MWCNT applications [50].

#### 4.2.3. Soil Microorganisms

##### Soil Microbial Diversity

In order to explore the effects of soil amendments on microbial communities more in depth, the relationships between carbon-containing amendments and microbial communities in the soil were examined. Our data showed that only when a high dose of ZBC was applied would the abundance and uniformity of soil microbial diversity be significantly reduced (Figure 3). It is known that organic substances (benzene and methoxyphenol) carried by biochar are not conducive to the growth of microorganisms. In this study, with the increase in MWCNTs, the ACE and Chao1 indices showed slight increasing trends, which were inconsistent with the results of previous studies. Possible reasons include the preparation method of the material, soil type, and other factors. For G, the number of OTUs and the ACE and Chao1 indices were lower than those for CK, indicating that the toxic effect of G on microorganisms was higher than that of MWCNTs. This was because G has a smaller diameter, a larger specific surface area, and stronger surface antimicrobial ability [51].

##### Soil Microbial Composition and Structure

The results showed that the dominant bacteria in the soil did not change after the application of G, MWCNTs, and ZBC and that microbes reacted differently to the varied carbonaceous materials. Acidobacteria prefer living in an acidic environment. In this study, the input of G and MWCNTs increased the RAs of Acidobacteria in the soil, while ZBC reduced their abundance. This suggested that the addition of G and MWCNTs enhanced the acidity of the soil, while ZBC increased soil alkalinity, which was consistent with the change trend observed for soil pH. Chloroflexi have a preference for extreme environments [52]. The RAs of Chloroflexus decreased with the application of G, MWCNTs, and ZBC, indicating that the soil environmental conditions were improved to some extent. Actinomycetes played a role in dissolving phosphorus, which could convert difficult-to-use inorganic phosphorus into phosphorus forms that facilitate absorption and utilization by crops, thereby increasing available phosphorus contents [53]. An increase in Actinomycetes was observed under ZBC treatment, which may explain the increase in AP. RDA analysis suggested that the input of too much ZBC (5%) would cause significant changes to the composition of the bacterial community in the soil, and a distinct dose effect on the soil microbial community structure was observed.

Correlation analysis showed that with the application of ZBC soil bacteria responded more strongly to soil enzyme activity and nutrients than with the application of G and MWCNTs (Table S3; Figure S6). Additionally, the relationships between soil enzyme activities, soil nutrients, and soil bacteria were not simple, but complex and dynamic. Therefore, more in-depth study is needed for a better understanding of the interactions among carbonaceous amendments, microbial communities, soil nutrients, and soil enzyme activities.

## 5. Conclusions

Natural and engineered carbonaceous amendments with different structures were selected to explore the impacts on soil cadmium fractions and soil microecology. Based on the results, we concluded that ZBC, G, and MWCNTs could effectively reduce the content of EX-Cd in soil and play a positive role in the maintenance of soil microecology. Correlation and RDA analyses showed that ZBC, G, and MWCNT treatments could increase contents of OM and AK, increase the activities of SUC and Urea, and regulate the abundance of bacteria to reduce the content of EX-Cd in soil. Additionally, the effect of ZBC on EX-Cd reduction was greater than the effects of G and MWCNTs because of ZBC’s stronger electronegativity, more abundant oxygen-containing functional groups, better regulation of the physical and chemical properties in the soil, and its strong ability to change soil microbial structure in a direction that was conducive to soil cadmium fixation.

## Supplementary Materials

The following supporting information can be downloaded at: https://www.mdpi.com/article/10.3390/ijerph191912381/s1, Figure S1: Effect of carbonaceous amendants on soil nutrient. (A) AN, alkali nitrogen; (B) AP, available phosphorus and (C) AK, available potassium. Figure S2: Effect of carbonaceous amendants on soil enzyme activity. (A) CAT, catalase enzyme; (B) Urea, urease enzyme; ACP, (C) acid phosphatase enzyme and (D) SUC, sucrase enzyme. Figure S3: Rarefaction curves. Figure S4: elative abundances of the top ten species with the highest abundances at the phylum level. Figure S5: Principal component analysis (PCoA) of soil bacterial community structure. Figure S6: Correlation between dominant microbial phyla, soil enzyme activity, and nutrients. Table S1: Physical and chemical properties of the tested soil. Table S2: Structure characteristics of carbonaceous amendants. Table S3: Analysis of correlation between microbial diversity, enzyme activity and soil nutrients. (* *p* < 0.05, ** *p* < 0.01, *** *p* < 0.001, and the same applies hereafter.) Table S4: Correlation analysis of physicochemical properties and Cd fractions of carbon-based porous materials. Table S5: Correlation analysis of soil physicochemical properties and Cd fractions.
