# Supplementary material for: Effects of Carbonaceous Materials with Different Structures on Cadmium Fractions and Microecology in Cadmium-Contaminated Soils
Source: Int J Environ Res Public Health. 2022 Sep 28;19(19):12381. doi: 10.3390/ijerph191912381 (PMC9564624; doi:10.3390/ijerph191912381)
Supplement: Supplementary file 1 [file ijerph-19-12381-s001.zip › ijerph-1880365-supplementary.pdf]

Table S1 Physical and chemical properties of the tested soil

moisture content (%)	pH	OM ($mg\cdot kg^{-1}$)	CEC ($cmol\cdot kg^{-1}$)	total Cd
3.08	6.16	34.43	7.13	3.76

Table S2 Structure characteristics of carbonaceous amendants

carbonaceous amendants	BET suface ($m^2\cdot g^{-1}$)	Pore volume ($cm^3\cdot g^{-1}$)	Pore diameter (nm)	Zeta potential
G	588.828	1.545	10.723	-6.78
MWCNTs	123.139	0.337	10.33	-6.17
ZBC	4.626	0.012	13.635	-30.93

Table S3 Analysis of correlation between microbial diversity, enzyme activity and soil nutrients(*:p<0.05, **:p<0.01, ***p<0.001, and the same applies hereafter.)

Treatment	Index	NMDS1	NMDS2	Chao1	Shannon
	Urea	-0.046	0.168	0.435	0.659
	ACP	0.028	0.269	0.135	0.331
	CAT	0.519	-0.787*	0.021	-0.326
G	SUC	-0.132	0.474	0.228	0.566
	AP	-0.014	0.343	0.311	0.501
	AK	0.014	0.314	0.211	0.381
	AN	-0.061	0.389	0.532	0.818**
	Urea	-0.281	-0.198	-0.081	-0.031
	ACP	0.170	-0.527	0.639	-0.495
	CAT	0.554	-0.361	0.202	-0.203
MWCNTs	SUC	0.373	-0.275	0.395	0.037
	AP	0.152	-0.572	0.421	-0.156
	AK	0.383	-0.237	0.188	0.051
	AN	-0.138	0.628	-0.471	0.104
	Urea	0.943**	-0.017	-0.774*	-0.876**
	ACP	-0.920**	0.103	0.754*	0.815**
	CAT	0.630	0.174	-0.434	-0.524
ZBC	SUC	0.944**	0.019	-0.820**	-0.889**
	AP	0.963**	-0.019	-0.870**	-0.969**
	AK	0.962**	-0.02	-0.831**	-0.917**
	AN	-0.870**	0.168	0.696*	0.860**

Table S4 Correlation analysis of physicochemical properties and Cd fractions of carbon-based porous materials

Index	EX-Cd	RD-Cd	OX-Cd	RS-Cd
BET	0.501	-0.832**	0.743**	-0.441
Pore volume	0.505	-0.836**	0.752**	-0.448
Pore diameter	-0.229	-0.308	0.580*	0.070
Zeta	0.755**	-0.721**	0.812**	-0.757**

Table S5 Correlation analysis of soil physicochemical properties and Cd fractions

Material	Index	EX-Cd	RD-Cd	OX-Cd	RS-Cd
G	pH	0.663	0.547	-0.357	-0.650
	CEC	-0.506	-0.448	0.589	0.389
	OM	-0.748*	-0.611	0.607	0.656
MWCNTs	pH	0.722*	0.608	-0.703*	-0.643
	CEC	0.365	0.374	-0.101	-0.451
	OM	-0.589	-0.621	0.551	0.599
ZBC	pH	-0.889**	-0.423*	0.015	0.919**
	CEC	-0.864**	-0.275	0.105	0.835**
	OM	-0.842**	-0.374	0.210	0.814**

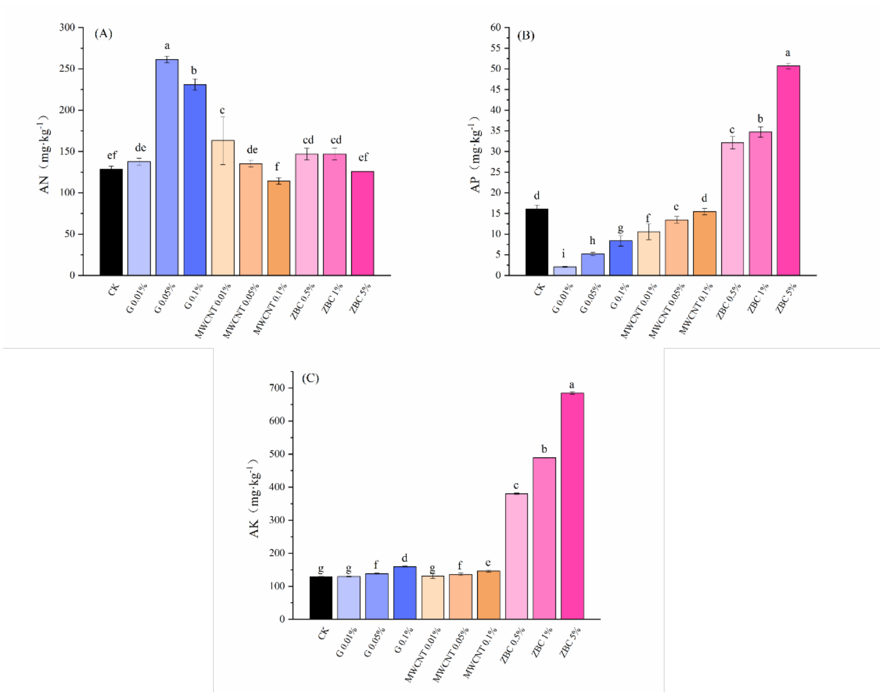

Figure S1. Effect of carbonaceous amendants on soil nutrient. (A) AN, alkali nitrogen; (B) AP, available phosphorus and (C) AK, available potassium.

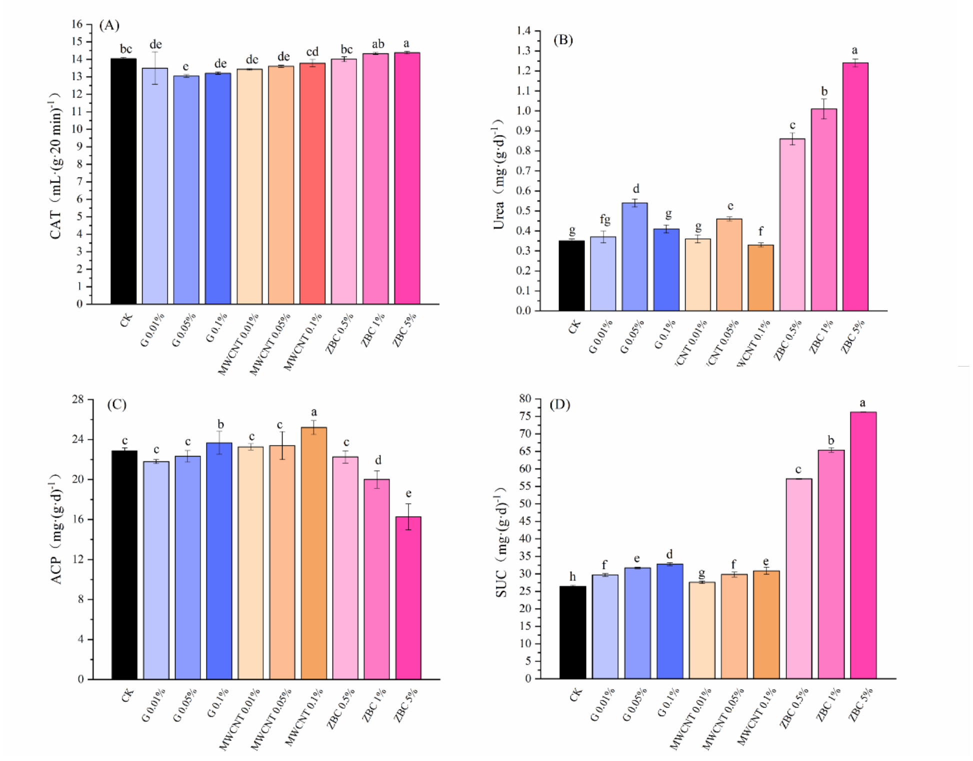

Figure S2. Effect of carbonaceous amendants on soil enzyme activity. (A) CAT, catalase enzyme; (B) Urea, urease enzyme; ACP, (C) acid phosphatase enzyme and (D) SUC, sucrase enzyme.

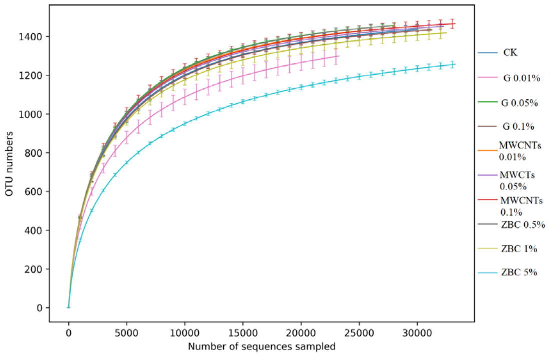

Figure S3. Rarefaction curves

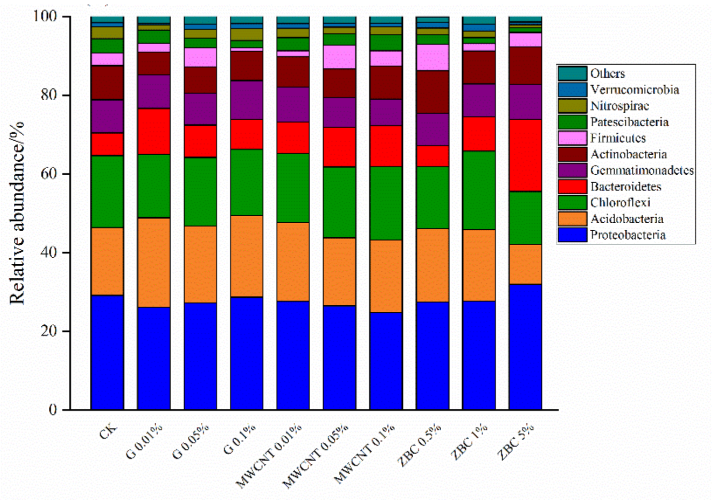

Figure S4. Relative abundances of the top ten species with the highest abundances at the phylum level

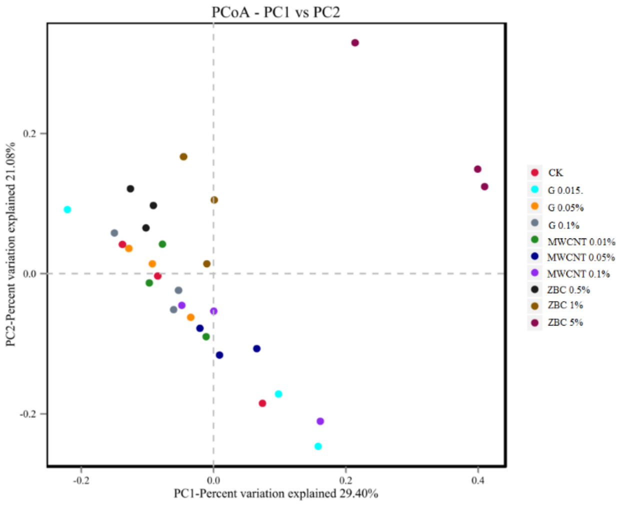

Figure S5. Principal component analysis (PCoA) of soil bacterial community structure

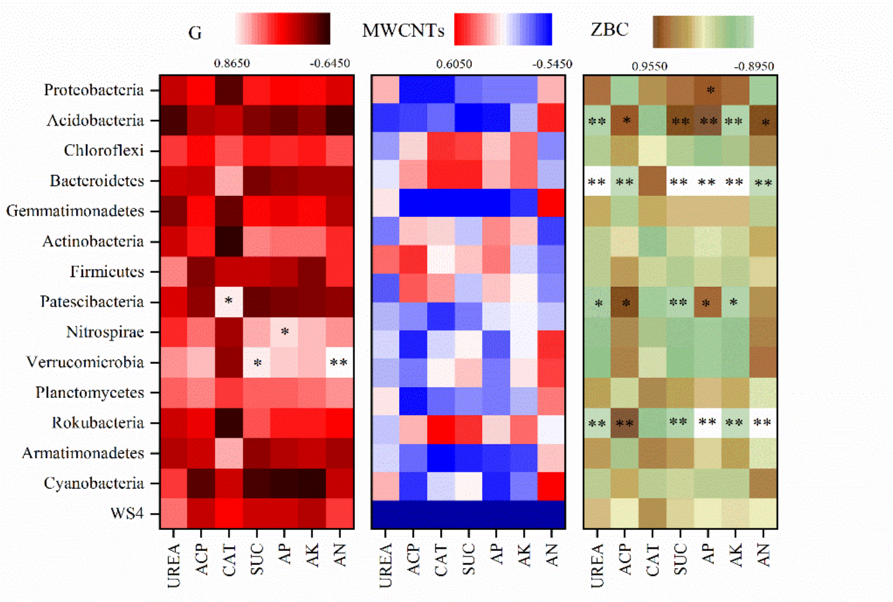

Figure S6. Correlation between dominant microbial phyla, soil enzyme activity, and nutrients
